# Data-Driven Detection of Subclinical Keratoconus via Semi-Supervised Clustering of Multidimensional Corneal Biomarkers

**DOI:** 10.1016/j.xops.2025.100998

**Published:** 2025-11-11

**Authors:** Lynn Kandakji, Shafi Balal, Aleksander Stupnicki, Siyin Liu, Marcello Leucci, Dan Gore, Bruce Allan, Nikolas Pontikos

**Affiliations:** 1 University College London Institute of Ophthalmology, 11-43 Bath Street, London EC1V 9EL, United Kingdom; 2 Moorfields Eye Hospital NHS Foundation Trust, London, United Kingdom; 3 University College London Medical School, 74 Huntley St, London WC1E 6DE, United Kingdom

**Keywords:** Keratoconus, Optical coherence tomography, Artificial intelligence, Machine learning, Cluster analysis

## Abstract

**Purpose:**

To objectively identify subclinical keratoconus (SKC) from a large sample of healthy and keratoconus (KC) patients via a data-driven framework on corneal imaging data from an anterior-segment OCT (AS-OCT) device (MS-39, CSO Italia).

**Design:**

A retrospective cohort study.

**Subjects:**

At 2 sites within the Moorfields Eye Hospital network in London, United Kingdom, 25 816 corneal scans from 5005 patients, including 3605 with KC and 1400 healthy control patients, were acquired between 2020 and 2024.

**Methods:**

Principal component analysis (PCA) followed by Gaussian mixture modeling (GMM) was applied to AS-OCT–derived data, including 20 KC indices and patient age, to identify SKC eyes, which were then statistically compared against healthy and KC eyes. Subclinical KC eyes were also validated against external systems including same-day Pentacam (Oculus Optikgeräte) scans, Belin-Ambrosio’s ABCD system, KC progression criteria determined by a panel of corneal specialists, and the Moorfields Corneal Cross-linking (CXL) Risk Calculator.

**Main Outcome Measures:**

Detection of SKC and progression of these eyes to clinically diagnosable KC over time.

**Results:**

The GMM identified 166 eyes from 161 patients with distinct structural differences between healthy and KC eyes. These eyes clustered in the morphometric transition zone in PCA space and were predominantly classified as ABCD stage 0. However, they demonstrated asymmetry with their fellow eye, higher predicted CXL risk at 1–4 years (*P* < 0.001), and faster progression to KC (log-rank *P* < 0.0001) compared with healthy eyes. Among SKC eyes with longitudinal data, 72.7% met Global Consensus criteria for progression.

**Conclusions:**

Subclinical KC remains challenging to detect, and although classic staging such as ABCD retains clinical utility, it is insufficient for early disease detection. Principal component analysis followed by GMM classification on a multidimensional AS-OCT dataset identifies a distinct and high-risk SKC group. This semisupervised framework offers a complementary tool for early risk stratification and can be applied to new patients via projection into the learned PCA space and computation of KC probability. Threshold values corresponding to the 25th and 75th percentiles of KC probability for each parameter may serve as clinical context for flagging eyes when multiple features fall in the atypical range.

**Financial Disclosures:**

Proprietary or commercial disclosure may be found in the Footnotes and Disclosures at the end of this article.

Early detection of keratoconus (KC), a progressive ectatic corneal disease, is critical to preventing irreversible visual decline and mitigating the risks associated with corneal refractive surgery.^1^, ^2^, ^3^ Missed or delayed diagnosis significantly increases the risk of postoperative iatrogenic keratectasia, a serious complication of corneal laser procedures.^4^^,^^5^ In this context, detection is increasingly essential because of the rising global prevalence of visually significant myopia, which is predicted to exceed 50% in many countries by 2050.^6^ When KC is identified early, interventions such as corneal collagen cross-linking (CXL) can halt disease progression in over 90% of cases,^7^^,^^8^ reducing the likelihood of corneal transplantation or future dependence on specialized contact lenses.^9^^,^^10^ Subclinical KC (SKC) is believed to represent the earliest detectable stage of disease, marked by subtle deviations in corneal morphology that are not readily identified using standard clinical tools.^11^^,^^12^

Despite its clinical relevance, there is no consensus on the definition and diagnostic criteria for SKC.^11^ The variability in use of terminology related to KC’s earliest stage, such as forme fruste, subclinical, early-stage, asymmetric, and KC suspect, has contributed to variability in diagnostic consistency and delayed intervention.^3^^,^^13^ A 2015 Delphi panel^14^ concluded that posterior elevation abnormalities must be present to diagnose SKC; however, their report did not provide specific data or references to support their agreement.^15^ Subsequent literature review found that posterior corneal surface metrics performed worse than anterior corneal and thickness metrics in differentiating SKC from normal controls.^16^ This ambiguity has led to circular logic in the validation of studies that use artificial intelligence methods to try and detect SKC. Researchers often define SKC using specific topographic or tomographic thresholds and then train machine learning models to detect those same criteria, an approach that may undermine the objectivity and generalizability of model evaluation. As a result, morphologic features used to define the subclinical cohort differ markedly across studies, limiting opportunities to compare findings and develop a unified diagnostic framework.^17^

High-resolution corneal imaging platforms such as the MS-39 (CSO Italia), a combined Placido and anterior-segment OCT (AS-OCT) tomographer, offer multimodal insights into corneal structure,^18^^,^^19^ but threshold-based classification remains insufficiently sensitive for capturing early, heterogenous morphological changes.^20^ Although supervised machine learning approaches have strong performance in detecting clinical stages of KC,^21^ these eyes are already identifiable through clinical examination. The clinical challenge lies in flagging eyes at risk of developing KC before overt structural or functional deterioration in the cornea occurs.^22^ In this study, we hypothesize that SKC represents a probabilistic intermediate state that can be identified between healthy and KC eyes through a data-driven approach without reliance on arbitrary thresholds.

## Methods

### Study Design and Ethics

This retrospective, observational study analyzed corneal imaging data collected during routine clinical care between 2020 and 2024 across 2 sites within the Moorfields Eye Hospital (MEH) network in London, UK.

Because this was an observational study using anonymized data collected in the course of routine clinical practice, individual patient consent was not required. This research was approved by the Institutional Review Board and the Ethics Committee of the UK Health Research Authority (Ref: 22/PR/0249). The study protocol was reviewed and approved by the Clinical Audit Assessment Committee of MEH National Health Service (NHS) Foundation Trust (reference CA17/CED/03). All research adhered to the tenets of the Declaration of Helsinki.

### Instrument and Feature Set

All data were obtained using the MS-39 AS-OCT system (CSO Italia), which combines Placido-disk corneal topography with low-coherence (840 nm) AS-OCT.^23^ The 2 are integrated via the proprietary Phoenix software (version 4.1.3) to produce comprehensive corneal maps, including curvature, elevation, and pachymetric measurements across both anterior and posterior corneal surfaces.^18^

Twenty device-derived corneal tomographic parameters relevant to KC detection were extracted from each scan. They are described in Table 1, along with normal and KC thresholds, which are provided to aid interpretation and should not be considered as definitive diagnostic cutoffs. Although none of these indices are diagnostic in isolation, they have demonstrated high reproducibility and discriminative performance across the KC disease spectrum^23^ and were used as the primary input for all analyses. Raw device outputs were exported in structured comma-separated values format for all scans, including the 20 KC indices and associated metadata. All analyses were performed using R version 4.3.1.

**Table 1:** Corneal Feature Definitions from MS-39

Feature	Unit	Definition	Normal Range
Symmetry index (SI)	mm	Difference in curvature or elevation between the superior and inferior cornea	±0.1
Center-surround index (CSI)	mm	Difference in curvature or elevation at the apex vs. the surrounding annular region	±0.1
Ectasia index (EI)	mm	Multiquadratic composite score of significant Zernike coefficients for corneal surface	0–0.5
Root mean square (RMS)	mm	Deviation between the corneal surface and a best-fit reference surface	≤0.02
Maximum keratometry (Kmax)	mm	Maximum Gaussian curvature (steepest point) of corneal surface	7.70–7.90
Delta Z (Δzmax)	μm	Maximum height of the bulging zone from the elevation vs. normality map	0–20
Notable Points Radius (NotablePtsR)	mm	Mean radial distance between 7 notable points on the cornea from their respective barycenter. These include the locations of minimum epithelial, stromal, and total corneal thickness; maximum anterior and posterior corneal curvature; and maximum anterior and posterior elevation.	>0.6
Thickness symmetry index (TSI)	%	Difference in corneal thickness between 2 symmetric hemi-corneas, usually across the vertical meridian	±10
Pattern deviation of thickness symmetry index (PD-TSI)	%	Compares the actual TSI distribution of the patient to a reference population of normal eyes	±10
% thickness index of the full cornea (PTI)	%	Expresses the proportion of the total corneal volume occupied by tissue thinner than the average	45–55
% epithelial thickness index (PEpiTI)	%	Relative contribution of the epithelial thickness to the total corneal thickness across the corneal surface and reflects epithelial compensation/remodelling	10
Minimum pachymetry (ThkMin)	μm	Value of the point with minimum total corneal thickness	500–600
Minimum stromal pachymetry (StrThkMin)	Μm	Value of the point with minimum total stromal thickness. The stromal thickness is less prone to remodeling than the epithelium and thus is a more stable indicator of true corneal structure.	470–550
Minimum epithelial pachymetry (EpiThkMin)	Μm	Value of the point with minimum total epithelial thickness	50

### Patient Cohorts

A total of 25 816 MS-39 scans were collected from 5005 patients. Two cohorts were defined.

• Keratoconus (KC) group: 12 501 scans from 3605 patients with clinician-assigned diagnosis of KC. Patients were identified using an structured query language-based query of the hospital’s data warehouse, targeting structured diagnostic fields in the electronic medical record. Selected patients, therefore, had a clinical label of KC that applied at the level of the patient rather than individual eyes. Postoperative cases (e.g., CXL and keratoplasty) were excluded at this stage through additional structured query language filters. All patients in this group were acquired at MEH NHS Foundation Trust.
• Control group: 13 315 scans from 1400 myopic individuals screened for refractive and lenticle extraction surgical screening. Eyes were deemed free of corneal pathology and ocular comorbidities following clinical evaluation by refractive surgeons. All patients in this group were acquired at Moorfields Private Hospital.

### Data Quality Control

Scan fidelity was assessed using 2 device-derived metrics: Placido/OCT coverage (OC) and section coverage (SC). These quantify the proportion of the corneal surface successfully captured by the Placido topography and AS-OCT subsystems, respectively,^24^ and account for common acquisition artifacts such as tear film instability, motion blur, and misalignment. To determine appropriate thresholds for scan inclusion, we evaluated the within-subject standard deviation of the KC indices across different OC and SC coverage levels. This reflects measurement repeatability, where higher values indicate lower consistency across repeated scans. Minimum thresholds for OC and SC were determined to ensure acceptable repeatability. OCT coverage was used as the primary quality metric, with SC serving as a secondary criterion for inclusion when OC was suboptimal. When multiple scans per eye were acquired on the same day, the scan with the highest composite quality was selected.

Each KC index was also reviewed for errant values outside physiologically plausible ranges, including nonpositive values for thickness parameters and keratometry readings and negative values for root mean square error and distance metrics. A detailed summary of exclusion thresholds is provided in Supplemental Table A; available at www.ophthalmologyscience.org. Additionally, an isolation forest algorithm was applied to detect extreme multivariate outliers. This method constructs an ensemble of decision trees that isolate individual points based on recursive partitioning, allowing for efficient anomaly detection without parametric assumptions.^25^

### Semisupervised Phenotype Classification

We hypothesized that SKC eyes are an intermediate morphological phenotype positioned between KC and structurally normal corneas. Given the absence of reliable ground truth labeling, we implemented a semisupervised framework to identify cases.

We leveraged our 2 labeled groups (healthy and KC) to learn the morphological extremes of the disease spectrum. The KC group was randomly downsampled to match the size of the healthy group, with 1400 patients in each group. Downsampling the KC group to match the healthy group mitigates the Gaussian mixture modeling (GMM) implementation from inferring class priors from the sample sizes and reduces overrepresentation of KC cases, which could reduce sensitivity to intermediate or borderline morphologies. For all included patients, only the earliest available scan per eye was retained to eliminate bias from disease progression. Dimensionality reduction was applied to the set of 20 corneal indices as well as patient age, for a total of 21 features. All features were z-score normalized. Principal component analysis (PCA) was used to decorrelate features and capture the dominant sources of variance and axis of disease severity. Principal component analysis is a method to linearly combine, as a weighted sum, many correlated measurements into a few independent axes that capture the main patterns of variation in the data. Each axis, called a principal component (PC), represents a single direction of variation in the data, with PC1 capturing the largest source of variation, PC2 the next largest, and so on. The top 2 PCs were retained for downstream analysis.

Two single-component GMMs were fit separately to the healthy and KC cohorts in the PC1-PC2 space using the expectation-maximization algorithm, assuming equal class priors. The PC1–PC2 space refers to a 2-dimensional plot of the first 2 PCs, where each axis represents a weighted sum of multiple corneal measurements, allowing overall patterns of variation between eyes to be visualized more clearly. This 2-component formulation reflects the prevailing clinical dichotomy of healthy versus KC. Models with >2 components were also tested but resulted in poorer overall fit and diverged from the data-driven intent of the analysis.

For any given eye x, the posterior probability of KC, P(KC|x), was computed using Bayes’ rule. In Bayesian terms, the *posterior* refers to the updated probability of a hypothesis after considering the observed data (here, the probability that an eye belongs to the KC distribution given its corneal features). To avoid confusion with the clinical use of “posterior” referring to the posterior corneal surface, we will hereafter refer to posterior KC probability simply as KC probability. Because the model only includes 2 diagnostic classes, the probability of being healthy is defined as P(Healthy|x)=1–P(KC|x). For clinical interpretation, these continuous probabilities were then converted into categorical labels using post hoc confidence intervals:

• Healthy: P(KC|x)<0.05 (i.e., P(Healthy|x)>0.95)
• Keratoconus: P(KC|x)>0.95
• Subclinical KC: 0.25≤P(KC|x)≤0.75 (equivalently 0.25≤P(Healthy|x)≤0.75, i.e., low confidence for either class)

Eyes outside the intermediate band but not exceeding 0.95 were assigned to the more likely class. Thus, SKC is not a third modeled cluster; it denotes cases near the decision boundary (where the Bayes factor is close to 1), reflecting ambiguity between healthy and KC. The 0.95 high-confidence threshold and the 0.25–0.75 intermediate band are standard, conservative probability thresholds used after density estimation to aid clinical interpretability without altering the fitted models.

### Batch Effects

To assess the presence of batch effects resulting from the use of different clinical sites, of which there was no patient overlap, we conducted a targeted validation using a prospectively recruited cohort of nine healthy patients who underwent bilateral imaging at both locations. Informed consent was obtained from all participants before data acquisition. All individuals were imaged on the same MS-39 AS-OCT device model, first at MEH NHS and then at Moorfields Private Hospital, with both scans performed within a 2-hour window. All imaging was conducted with identical calibration, positioning, lighting, and acquisition protocols to ensure that any observed differences were attributable solely to site- or device-related factors.

For each patient, paired scans from both sites were projected into PCA space using the transformation derived from the main cohort. We computed the Euclidean distances in PCA space between the 2 sites using PC1 and PC2, which served as a proxy for multivariate deviation in corneal metrics. To statistically evaluate whether these intersite distances reflected systematic site differences, we performed a 1-sample *t* test comparing the distribution of distances to a null hypothesis of zero mean displacement.

### Clinical Validation and Utility

To evaluate the validity and prognostic value of the SKC phenotype, we conducted a series of retrospective analyses. This included 7658 scans from 1827 patients with at least 1 follow-up spanning up to 2 years.

#### Longitudinal Changes in Disease Likelihood

We applied the previously trained PCA–GMM model to eyes from follow-up visits that were not included in the initial model. Principal component analysis transformation was applied to follow-up scans using the loading matrix derived from the training set, and KC probability was computed using the fixed GMM model parameters, ensuring that longitudinal analyses reflected projection into the same morphological space.

Structural progression in SKC eyes was defined using the 2015 Global Consensus definition as a change greater than expected measurement noise for the imaging device used in ≥2 parameters between visits. We derived the MS-39 thresholds based on Seiler et al.^26^

(A) Steepest anterior curvature radius decrease >0.1 mm
(B) Steepest posterior curvature radius decrease >0.05 mm
(C) Minimum pachymetry reduction ≥20 μm

#### CXL Risk Stratification

To assess the clinical risk profile of eyes identified as SKC, we integrated structural imaging data with the Moorfields CXL Risk Calculator (https://beta.moorfieldscxl.com), a peer-reviewed external prognostic tool.^27^ Risk scores were generated via batch submission to the calculator’s backend Application Programming Interface using age, Kmax, Front K1, and minimum pachymetry from MS-39 scans. The calculator outputs a probability score (0–1) indicating the likelihood that an eye will require CXL within a given time horizon, based on statistical patterns learned from demographic and serial Pentacam HR (Oculus Optikgeräte) corneal tomography data from 8701 eyes of 4823 patients with early to mild KC. When multiple eligible scans existed for a patient, the earliest one where the patient was classed as SKC was used. Healthy eyes were matched similarly using their earliest available scan.

Predicted CXL risk probabilities after 1, 2, 3, and 4 years were extracted for each eye, and SKC eyes were compared with healthy using pairwise Wilcoxon rank-sum tests. For eyes with at least 1 follow-up, we calculated the change in predicted risk between consecutive visits.

#### Survival Analysis of Time to Keratoconus Conversion

A Kaplan–Meier (KM) survival model was constructed to compare time to progression between SKC and healthy eyes. Progression was defined as a subsequent reclassification to KC at any follow-up visit. Time-to-event was measured in days between the baseline visit and either the date of conversion or the last available follow-up. Right-censoring was applied to nonprogressors. In these instances, eyes were considered “at risk” until their last follow-up, after which their subsequent clinical status remained unknown. This approach ensures unbiased risk estimation by incorporating both converted and nonconverted eyes, rather than assuming nonconversion equates to permanent stability. Although the KM method provides an unadjusted estimate and visualization of progression risk, to address potential confounding by age, we performed 1:1 nearest-neighbor matching of healthy to SKC eyes on baseline age, without replacement, before survival analysis. This ensured that differences in progression risk were not attributable to baseline age imbalances.

#### Cross-Sectional Agreement between GMM and ABCD Staging

Gaussian mixture model labels were compared with ABCD staging scores calculated from Pentacam scans taken the same day. ABCD staging was computed using the steepest anterior K value (A), steepest posterior K value (B), minimum pachymetry (C), and best-corrected visual acuity (D). A composite ABCD score was assigned based on the most frequent stage among components A–D.

## Results

### Scan Repeatability and Quality Thresholds

Within-subject standard deviation of KC indices increases markedly when either OC or SC falls below 65% for PC and 85% for SC, indicating reduced measurement repeatability. Repeatability stabilized at OC values between 60% and 64% and remains consistent as coverage increases, as seen in Supplemental Figure A; available at www.ophthalmologyscience.org. In contrast, SC demonstrated greater variability across the coverage range, with acceptable repeatability only observed at 85%–95%, and lower overall consistency compared with OC. These findings indicate that OC is a more reliable metric of scan quality than SC. Quality filtering was performed in a hierarchical manner: scans were included if OC >65%; if OC was below this threshold, inclusion was still permitted if SC >85%.

**Figure 1:**
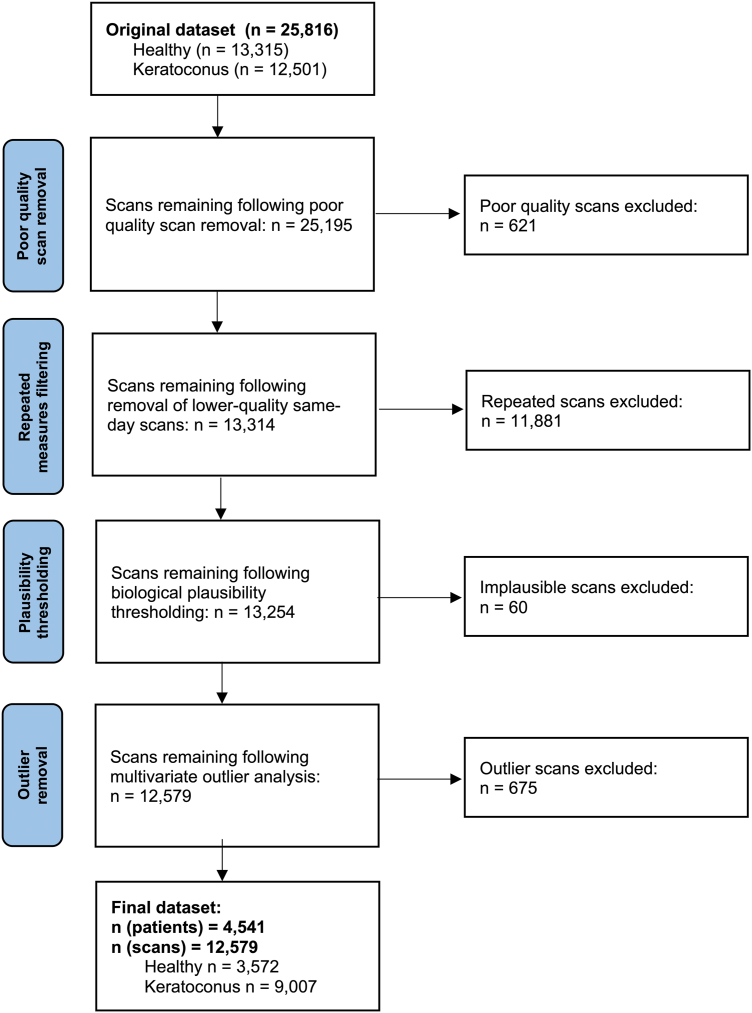
Flowchart illustrating dataset clean-up with the results of each preprocessing step.

After exclusion of scans that failed this quality criteria and removal of same-day repeated scans, biologically implausible values, and multivariate outliers, 48.7% of the dataset was deemed of good quality for further analysis. The impact of each step is detailed in Figure 1.

### Dataset Demographics

This study utilized 2 separate datasets for analyses. The majority of analyses used a large retrospective cohort, whereas analysis of batch effects used a small internal set of controls. Table 2 summarizes each dataset’s characteristics. The age and sex distributions of the downsampled KC group were not significantly different from those of the full KC cohort.

**Table 2:** Summary of Demographic Characteristics for the Retrospective and Prospective Cohorts

Characteristic	Retrospective Dataset	Prospective Dataset
Total eyes (n)	12 579	18
Total patients (n)	4541	9
Healthy (%)	28.4	100
Keratoconus (%)	71.6	0
Age (yrs)	33.6 ± 11.7 (range: 6–97)	27.2 ± 4.3 (range: 20–34)
Males (%)	60.9	44.4
Reported ethnicity (%)	34.7	100
White	31.0	44.4
Middle Eastern	24.0	11.1
South Asian	21.0	11.1
Black	15.3	0
East Asian	6.5	33.4
Mixed	2.2	0

Percentages are reported at the patient level.

### Dimensionality Reduction and Assessment of Intersite Variability

Principal component analysis was conducted on 4876 eyes from 3278 patients. This comprised 2438 eyes labeled as healthy and 2438 eyes labeled as KC. The first 2 PCs explained 78.0% of the total variance, with PC1 accounting for 71.7% and PC2 for 6.3%, as summarized in Supplemental Table B, available at www.ophthalmologyscience.org. Inspection of the scree plot (elbow method)^28^ confirmed that variance contributions declined sharply after PC2 (Supplementary Figure B; available at www.ophthalmologyscience.org), with higher-order PCs each explaining <2% of variance and largely reflecting measurement noise or patient-specific variation.

Each scan pair clustered tightly and mapped to adjacent positions, as seen in Supplemental Figure B, indicating high morphological concordance across sites. The mean Euclidean distance between site-paired scans was 0.12 ± 0.05, and no statistically significant difference from 0 was detected (*t* test, *P* = 0.368). Based on this, no batch correction was applied to the dataset.

### Keratoconus Severity Continuum

**Figure 2:**
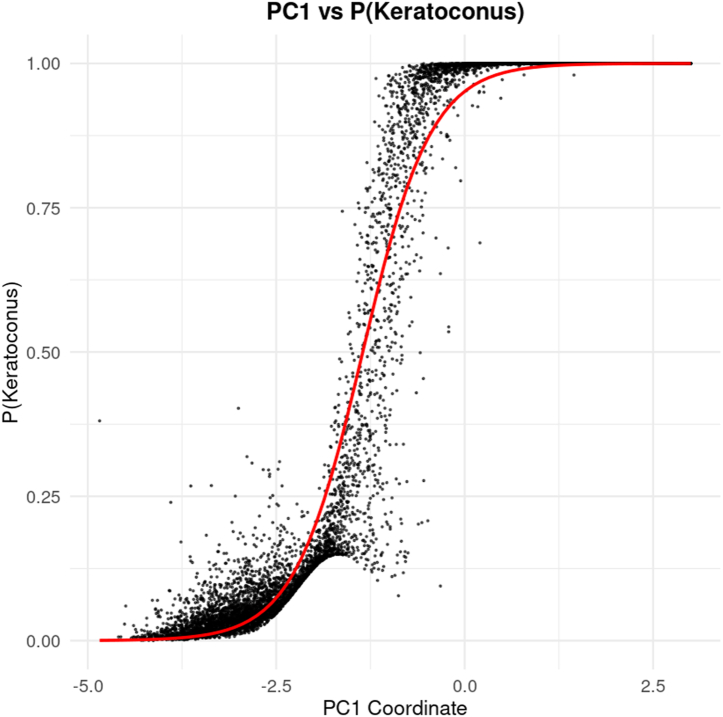
Gaussian mixture model (GMM)–derived P(Keratoconus) plotted against the PC1. There is a strong relationship observed between PC1 and P(Keratoconus), with increasing values along the x-axis (PC1) corresponding to greater disease severity. Each point represents a single eye, and the red curve indicates the fitted logarithmic trend line. The clear association between PC1 and keratoconus probability highlights that subtle corneal shape variations captured by unsupervised analysis align closely with disease severity. PC1 = first principal component; P(Keratoconus) = probability of keratoconus.

**Figure 3:**
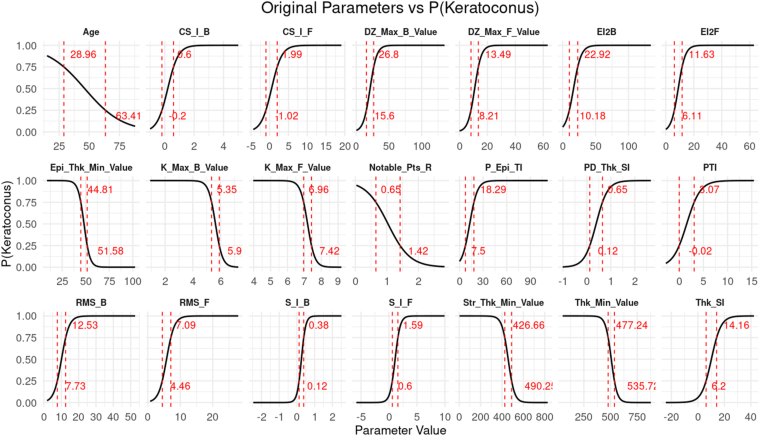
Smoothed curves showing how individual tomographic parameters relate to the Gaussian mixture model–derived P(Keratoconus). Each panel corresponds to 1 tomographic feature. Red dotted vertical lines mark the parameter values associated with 25% and 75% P(Keratoconus), with annotations indicating the exact values. These parameter–probability curves identify threshold ranges where subtle tomographic changes signal elevated keratoconus risk. P(Keratoconus) = probability of keratoconus.

Principal component 1 represented a continuous axis of KC severity, with increasing positive coordinates corresponding to a higher probability of KC (Fig 2). High PC1 loadings were observed for anterior and posterior surface indices, elevation deviations, and curvature-based parameters, whereas PC2 captured more localized variation, primarily reflecting age-related structural asymmetries and corneal thinning, as presented in Supplemental Figure C; available at www.ophthalmologyscience.org. Smoothed relationships between PC1 and each original parameter reveal inflection zones where subtle changes in certain indices, particularly stromal thickness, root mean square, and elevation metrics, translate to sharp increases in KC probability (Fig 3).

### Subclinical Group Reclassification

**Figure 4:**
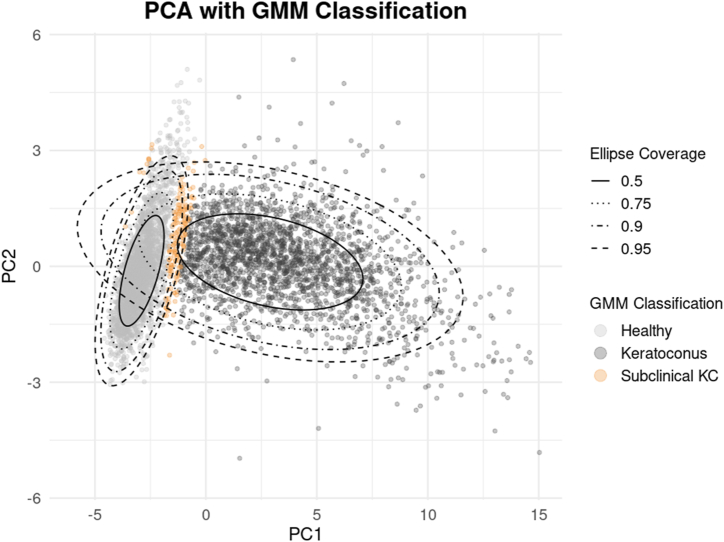
Classification of subclinical keratoconus using a 2-component GMM. Each point represents a single eye plotted according to a reduced set of 21 tomographic features using principal component analysis. The shaded gray ellipses indicate the regions where healthy eyes (light gray) and KC eyes (dark gray) are most likely to be located, with boundaries drawn at 50%, 75%, 90%, and 95% confidence levels. Eyes shown in orange fall outside the high-confidence regions of both groups and are labeled as subclinical KC. The model isolates eyes with ambiguous morphology, highlighting cases that may warrant closer monitoring despite not fitting cleanly into healthy or diseased categories. GMM = Gaussian mixture model; PCA = principal component analysis; KC = keratoconus.

Using the KC probability derived from the 2-component GMM, 166 eyes from 161 patients were identified as SKC from the PCA data. These included 50 eyes from the healthy cohort and 116 from the KC cohort. These eyes were not situated within the high-density cores of either group but overlapped with the low-probability tails of the healthy and KC distributions (Fig 4). When the probability thresholds were varied to 0.30–0.70 and 0.20–0.80, the number of eyes classified as SKC shifted slightly, to 162 and 168 eyes, respectively, corresponding to a relative change of <3%. Over 96.8% of eyes retained their original classification across thresholds. Statistically significant differences were observed between SKC and both healthy and KC eyes for all parameters, except for age between SKC and KC (Table 3). Subclinical keratoconus often presented unilaterally or asymmetrically, with only 9.6% of eyes showing bilateral SKC, whereas healthy (94.9%) and keratoconic (84.1%) eyes exhibited more symmetric fellow eye classifications. Subclinical KC was commonly associated with either a keratoconic fellow eye (54.6%) or a healthy fellow eye (42.5%).

**Table 3:** Comparison of 21 Features across Gaussian Mixture Model–Defined Groups of Healthy (p_KC_ <0.25), Subclinical Keratoconus (0.25 < p_KC_ < 0.75), and Keratoconus (p_KC_ >0.75) Groups

Metric (B = PosteriorF = Anterior)	Healthy		*P* Value	Subclinical Keratoconus		*P* Value	Keratoconus	
	Mean	Standard Deviation		Mean	Standard Deviation		Mean	Standard Deviation
Age	40.18	25.58	1.4e-35	31.39	11.07	0.299	30.76	7.99
SI B	0.03	0.12	4.3e-21	0.23	0.19	8.3e-122	1.34	0.76
SI F	0.30	0.49	1.9e-08	0.98	0.66	9.7e-120	5.10	3.07
CSI B	0.18	0.13	4.3e-191	0.25	0.26	4.9e-290	1.11	0.99
CSI F	0.51	0.77	8.0e-146	0.69	0.97	5.3e-275	3.57	3.50
EI B	4.19	4.45	2.9e-182	15.93	7.81	2.6e-289	75.45	38.23
EI F	3.60	2.66	2.2e-139	8.21	3.78	2.3e-282	34.55	18.32
RMS B	5.86	2.69	2.3e-17	10.14	5.47	2.4e-163	30.28	31.80
RMS F	3.63	1.63	3.5e-129	5.52	1.83	4.3e-229	15.77	7.62
KMax B	6.11	0.39	5.6e-71	5.59	0.57	1.3e-220	4.41	0.68
KMax F	7.52	0.34	5.3e-84	7.21	0.40	1.3e-120	6.26	0.62
Δzmax B	9.61	4.88	2.6e-96	20.59	10.46	7.8e-110	77.97	57.25
Δzmax F	5.91	3.04	1.7e-94	10.38	3.86	5.7e-224	35.16	18.05
NotablePtsR	1.34	0.53	2.9e-23	0.82	0.60	4.4e-217	0.37	0.37
TSI	5.94	4.68	8.0e-150	10.76	5.04	8.2e-263	24.96	11.48
PD-TSI	0.18	0.38	7.2e-120	0.55	0.33	3.5e-263	1.03	0.49
PTI	0.18	2.48	1.6e-146	1.53	2.00	3.0e-267	5.87	2.78
PEpiTI	8.85	5.92	2.5e-124	11.82	9.46	1.7e-265	34.42	21.67
ThkMin	519.73	42.62	5.0e-58	488.46	34.90	3.5e-66	450.82	41.46
StrThkMin	465.79	43.23	7.1e-66	436.95	35.12	1.6e-88	405.58	40.41
EpiThkMin	49.56	3.91	5.5e-95	48.30	4.77	3.6e-196	41.24	5.44

*P* value is calculated between adjacent column groups.

### Subclinical Phenotype as a High-Risk Intermediate State

Among 1827 patients with at least 1 follow-up in a period of up to 800 days (approximately 2 years), 437 contributed 1 eye and 1390 contributed both, yielding 3217 eyes included in the longitudinal analysis. Based on GMM-derived classifications, 250 patients were consistently labeled as healthy, 1246 as KC, and 12 as SKC. The remaining 319 patients received more than 1 classification across visits, 190 of which were classified as SKC during at least 1 visit. Follow-up intensity was similar between groups: healthy eyes had 2.3 ± 1.5 visits per year, compared with 3.2 ± 1.3 visits per year for SKC eyes (*P* = 0.344 for visit count; *P* = 0.410 for follow-up duration).

**Figure 5:**
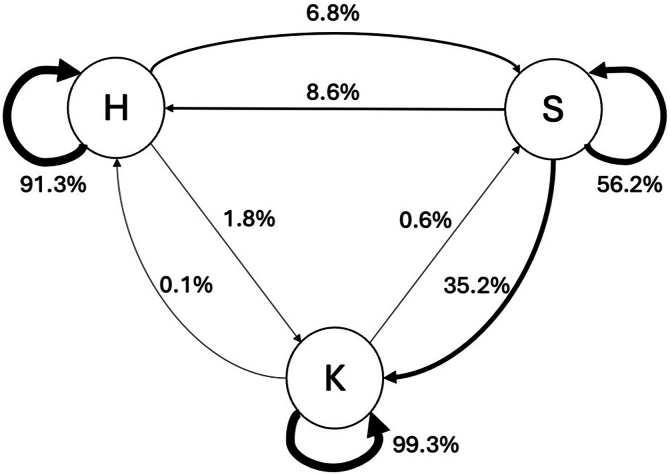
State diagram showing how eyes changed classification over time, across follow-up visits spanning up to 800 days after the first visit. Circles (nodes) represent the 3 disease states: healthy (H), subclinical keratoconus (S), and keratoconus (K). Arrows (edges) indicate observed transitions between states, with thicker arrows corresponding to higher transition probabilities. Most progression occurred from subclinical keratoconus to keratoconus, whereas direct transitions from healthy to keratoconus were rare.

Transitions between states were summarized using a first-order Markov model (Fig 5). Over 90% of healthy and KC eyes retain their initial classifications over time. In contrast, SKC eyes show instability, with only 56.2% retaining their classification and 35.2% transitioning to KC within an average follow-up period of 13 ± 4.5 months. Of the 8.6% of eyes reclassified back to normal, this occurred within 4.3 ± 2.1 months on average.

To further examine progression patterns, we visualized the longitudinal trajectory of KC component probabilities. Eyes initially classified as healthy exhibited minimal changes in KC probability over time, though a small subset showed sudden increases, as illustrated in Supplemental Figure D, available at www.ophthalmologyscience.org. In contrast, SKC eyes demonstrated a steady and gradual increase in KC probability across visits, supporting a progression toward disease conversion. This trajectory was primarily driven, in the following order, by thickness changes (minimum stromal thickness, minimum corneal thickness, and percentage thickness index), maximum anterior and posterior elevation, anterior root mean square, mean radius of notable points, posterior symmetry index, and posterior ectasia index, as shown in Table 4. These parameters were not used to define SKC at baseline; rather, they represent the structural features that significantly drove progression from SKC to KC. Changes in other indices over time were not statistically significant between groups. Although some of these metrics covary, as shown by Supplemental Figure E; available at www.ophthalmologyscience.org, it highlights the specific anatomy and parameters that are most sensitive to early disease changes.

**Table 4:** Statistical Significance of MS-39 Indices Associated with Early Keratoconus Progression, Defined as Transition from Gaussian Mixture Model (GMM)-Classified Subclinical Keratoconus (0.25<p_KC_<0.75) to Keratoconus (p_KC_>0.75)

Metric	D	Log_2_FC (95% CI)	*P* Value
Minimum stromal pachymetry (StrThkMin)	**↓**	**–0.45 (95% CI: –0.60to–0.30)**	**3.7 × 10^–11^**
Minimum pachymetry (ThkMin)	**↓**	**–0.40 (95% CI: –0.55to–0.25)**	**1.7 × 10^–8^**
Anterior delta Z (Δzmax_F)	**↑**	**0.80 (95% CI: 0.60to1.00)**	**7.9 × 10^–7^**
% thickness index of the full cornea (PTI)	**↑**	**0.35 (95% CI: 0.20to0.50)**	**3.5 × 10^–6^**
Anterior root mean square (RMS_F)	**↑**	**0.65 (95% CI: 0.45to0.85)**	**5.0 × 10^–5^**
Notable Points Radius (NotablePtsR)	**↓**	**–0.30 (95% CI: –0.45to–0.15)**	**2.0 × 10^–4^**
Posterior symmetry index (SI_B)	**↑**	**0.25 (95% CI: 0.10to0.40)**	**1.5 × 10^–3^**
Posterior ectasia index (EI_B)	**↑**	**0.50 (95% CI: 0.30to0.70)**	**2.4 × 10^–3^**
Posterior delta Z (Δzmax_B)	**↑**	**0.75 (95% CI: 0.50to1.00)**	**3.7 × 10^–3^**
Pattern deviation of TSI (PD-TSI)	**↑**	**0.30 (95% CI: 0.15to0.45)**	**3.8 × 10^–3^**
Anterior center-surround index (CSI_F)	↑	0.20 (95% CI: 0.05 to 0.35)	2.1 × 10^–2^
% epithelial thickness index (PEpiTI)	↑	0.40 (95% CI: 0.20 to 0.60)	6.7 × 10^–2^
Thickness symmetry index (TSI)	↓	–0.00 (95% CI: –0.01 to 0.01)	7.4 × 10^–2^
Posterior center-surround index (CSI_B)	↑	0.01 (95% CI: 0.00 to 0.02)	1.4 × 10^–1^
Posterior root mean square (RMS_B)	↓	–0.01 (95% CI: –0.05 to 0.01)	2.2 × 10^–1^
Minimum epithelial pachymetry (EpiThkMin)	↓	–0.00 (95% CI: –0.01 to 0.01)	5.2 × 10^–1^
Anterior symmetry index (SI_F)	↓	–0.07 (95% CI: –0.10 to –0.04)	6.2 × 10^–1^
Anterior maximum keratometry (Kmax_F)	↑	0.00 (95% CI: –0.01 to 0.01)	6.5 × 10^–1^
Posterior maximum keratometry (Kmax_B)	↓	–0.02 (95% CI: –0.02 to –0.02)	6.9 × 10^–1^
Anterior ectasia index (EI_F)	↓	–0.02 (95% CI: –0.10 to 0.02)	7.6 × 10^–1^

Effect size is reported as log fold-change (progressors vs nonprogressors) with 95% CI. Metrics are ranked by ascending *P* value, with smaller values indicating stronger discriminatory power. Bolded rows indicate statistically significant parameters for progression.

D = direction of effect (**↓**=decreasing, **↑**=increasing); log_2_FC = log of fold change; CI = confidence interval.

These findings were corroborated against progression criteria outlined in an expert opinion piece authored by a panel of corneal specialists.^14^ Among eyes classified as SKC, 72.7% showed evidence of progression. Of these, 54.4% met criterion A (anterior curvature), 67.0% met criterion B (posterior curvature), and 87.5% met criterion C (pachymetric thinning).

**Figure 6:**
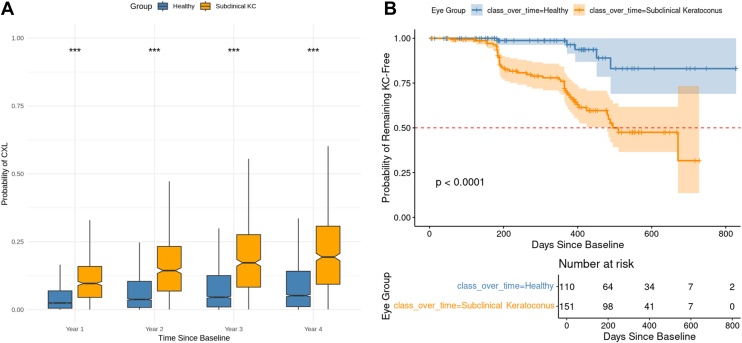
(**A**) Boxplots showing the predicted probability of requiring CXL at years 1 through 4, stratified by baseline classification using the GMM into healthy, subclinical KC, and KC groups. Asterisks denote statistically significant differences (∗∗∗*P* < 0.0001). (**B**) Kaplan–Meier survival curves estimating the probability of remaining KC-free over time for eyes classified at baseline as subclinical KC (orange) or healthy (blue). Shaded regions show 95% confidence intervals, and vertical tick marks indicate censored observations (eyes lost to follow-up). The red dashed line marks the 50% probability threshold. Eyes classified as subclinical KC at baseline had significantly higher predicted CXL risk and a steeper decline in KC-free survival compared with healthy eyes. CXL = corneal cross-linking; KC = keratoconus; GMM = Gaussian mixture model.

Subclinical keratoconus eyes also exhibited a significantly higher probability of requiring CXL in the future compared with healthy eyes (*P* < 0.001; Fig 6A), with risk increasing by an average of 10.2% for SKC eyes with follow-up and 4.06% for healthy eyes. Kaplan–Meier survival analysis, after age matching (mean baseline age 31.9 ± 12.1 years in both groups, n = 337 per group), revealed that SKC eyes had a substantially lower KC-free survival rate than healthy eyes (log-rank *P* < 0.0001). Approximately 500 days after the first visit, the probability of remaining KC-free had fallen below 50% for SKC eyes, whereas healthy eyes remained largely stable throughout follow-up (Fig 6B).

### GMM Captures Early Risk Patterns Missed by Belin–Ambrosio’s ABCD

**Figure 7:**
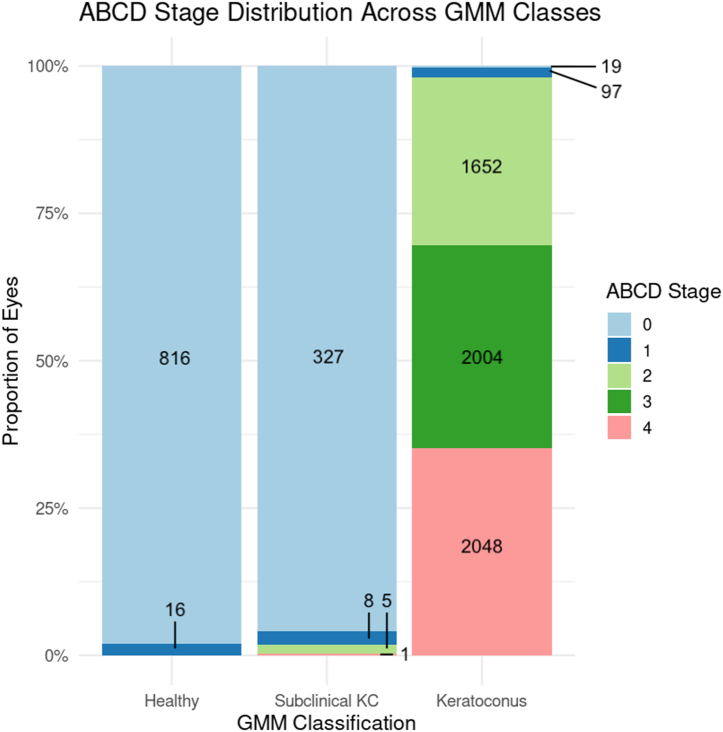
Distribution of ABCD KC stages at baseline, stratified by GMM-based classification into healthy, subclinical KC, and KC groups. Bars show the proportion of eyes in each ABCD stage (0–IV) within each group. Eyes classified as healthy were almost entirely stage 0, subclinical KC eyes clustered in stages 0–I, and KC eyes spanned the full spectrum up to stage IV, confirming that the GMM groupings aligned with increasing clinical disease severity. KC = keratoconus; GMM = Gaussian mixture model.

For 2857 patients in the healthy and KC groups with same-day Pentacam scans, 98.1% of GMM-classified healthy eyes and 95.9% of SKC eyes were assigned stage 0 on the ABCD grading system (Fig 7). Keratoconus eyes were predominantly distributed across ABCD stages 2 to 4. Among the 185 SKC eyes with at least 1 follow-up visit, 96.2% (n = 178) remained at stage 0. Of the 7 eyes that progressed, 3 advanced to stage 1, 3 advanced to stage 2, and 1 advanced to stage 4. When disaggregated by individual ABCD components, progression was most frequently observed in parameter D (best-corrected visual acuity), followed by C, A, and B. Pachymetry was the most frequent structural progressors under ABCD criteria, aligning with Global Consensus thresholds.

## Discussion

The identification of KC at the earliest stage remains one of the most pressing challenges in corneal diagnostics. This difficulty stems not only from the subtlety of the phenotype but also from the absence of a universal standard definition. Across studies and populations, reported prevalence estimates for KC range from under 0.1% to nearly 9%,^29^ a disparity driven in part by different diagnostic cutoffs and whether subclinical cases are included. A recent systematic review on SKC by Randleman et al found that many studies used subclinical group inclusion criteria that would not exclude clinical KC eyes.^16^ Even within a single cohort, the use of different cutoff values for diagnostic indices can dramatically shift classification.^16^^,^^30^ To date, none have attempted to distinguish normal from early KC without such assumptions.

The findings of this study directly address this gap by demonstrating that a semisupervised machine learning approach using GMM in a reduced feature space can identify a subgroup of morphologically atypical eyes not captured by traditional grading systems. Unlike fully unsupervised clustering, this approach ensures that the learned latent structure is anchored to clinically meaningful binary groups (0 = healthy; 1 = KC). The strength of this approach is that the “subclinical” group emerges from a region of maximal overlap within a biologically plausible continuum, rather than through arbitrary cutoffs. Within a 0.25 to 0.75 confidence threshold, the subclinical phenotype presents within a narrow biometric window, with thickness differences <65 μm, radius differences <0.5 mm, and elevation/ectasia index variations <1.

Overall, the eyes in this group demonstrated.

1. Almost total agreement with Belin-Ambrosio’s ABCD stage 0;
2. Significant structural differences from healthy and KC eyes;
3. Significant asymmetry with fellow contralateral eye; and
4. Consistent structural degradation over time

Although the ABCD display is the current standard for detecting KC and monitoring disease progression, our results suggest that its sensitivity to the detection of early disease is limited. Healthy and SKC eyes are largely indistinguishable on this scale, with both typically classified as stage 0 despite significant differences in structure. Progression is also not well predicted by ABCD staging but is reasonably predicted by the Gomes et al criteria. Only 3.8% of SKC eyes had progressed, and interestingly, this progression was mostly driven by functional decline (parameter D and visual acuity) rather than by anterior or posterior curvature or pachymetry (parameters A, B, and C). This is markedly lower than the results of the Gomes et al progression criteria, which indicated that 70% of SKC eyes had progressed, aligning with previous studies on SKC progression rates.^31^^,^^32^ Using GMM-derived KC probability, over 30% reached 75% probability of KC within 2 years. Subclinical keratoconus eyes also demonstrated a higher risk of requiring CXL within 4 years and shorter KC-free survival in Kaplan–Meier analysis.

This was driven specifically by corneal thickness changes, especially in the stroma, and by subtle elevations and irregularities in the anterior and posterior curvature. These changes, although not always sufficient to shift ABCD staging, may signal early biomechanical instability and help explain why some SKC eyes progress despite appearing clinically normal. Progression in anterior and posterior Kmax was not found to be statistically significant between healthy and SKC eyes and explained only 50% to 60% of eyes meeting the Global Consensus criteria, highlighting the limitations of traditional KC metrics in detecting early stages of the disease. Thickness changes were observed in >80% of SKC eyes, particularly at the stroma level. This supports prior findings that stromal metrics are more effective than epithelial metrics—which were not found significant in our cohort—in distinguishing SKC from healthy eyes.^2^^,^^33^ Some of these significant parameters have previously been linked to SKC in earlier studies.^16^ Our findings expand this understanding by showing that these features not only differ at baseline but are also associated with longitudinal progression.

The concept of subclinical KC as an intermediate state between healthy and KC has long informed topographic indices such as the Keratometry-Inferior-Superior-Astigmatism percentage index (KISA%)^34^ and the Cone Location and Magnitude Index (CLMI/CLMI.X).^35^ Although these indices reliably distinguish manifest KC from normal eyes, their performance in *subclinical or suspect eyes* has been weaker, owing to several factors. KISA% relied on only 4 anterior topographic parameters and applied rigid thresholds (≥100%), leading to substantial overlap between normal and KC suspects. CLMI.X incorporated cone location and magnitude, as well as pachymetric features, but still categorized eyes via fixed cutoffs. Although the recent CLMIX-AI^36^ adaptation leverages machine learning, published evaluations show that sensitivity and specificity remain consistently lower for KC suspects than for manifest KC.

Our framework differs in 3 critical ways. First, it uses a broad multidimensional feature set (20 tomographic indices plus age) encompassing anterior, posterior, elevation, and thickness parameters. Second, it models classification probabilistically: GMM yields a KC probability, with an explicit “gray zone” (0.25–0.75) rather than a binary or trinary cutoff. This reflects diagnostic uncertainty rather than masking it. Third, we benchmarked the subclinical group against external validators (ABCD staging, Gomes et al progression criteria, Pentacam tomography, and the Moorfields CXL Risk Calculator) and demonstrated longitudinal predictive value (higher CXL risk and faster conversion). These results show that the intermediate group identified is not an artifact of thresholding but represents a clinically meaningful high-risk state.

However, there are some limitations. The thresholds proposed in the 2015 article by Gomes et al remain insufficiently validated. The ABCD grading system was introduced, in part, in response to this consensus and provided 95% confidence limits for the true change in these parameters based on data from 252 normal (“early keratoconus”) and KC (“established disease”) patients. Further work should integrate adaptive thresholding for progression^37^ and investigate the metrics highlighted here to further refine early disease progression, which may be distinct from those needed for initial detection. Adaptive thresholding may also help mitigate measurement variability near the threshold of detectability—reflected by a small subset of SKC eyes that were reclassified as healthy within a short follow-up period. Although Kaplan–Meier models provide unadjusted estimates of progression risk and cannot simultaneously account for multiple baseline covariates (e.g., pachymetry or intereye asymmetry), we minimized the influence of age by matching SKC and healthy groups on baseline age prior to analysis. The results should therefore be interpreted descriptively. The consistency of Kaplan–Meier findings with independent validation further supports that the elevated risk observed in SKC eyes reflects a true biological signal rather than an artifact of unadjusted modeling. Moreover, our findings can only be applied directly to MS-39 corneal tomography. Although the indices used are broadly comparable across devices and were validated with same-day Pentacam scans, further external validation will be essential before generalizing this approach.

A further consideration is the proportion of scans excluded during quality control. In total, 48.7% of acquisitions were excluded. Of these, 94.5% were repeated same-day scans from the same eye, and 0.06% were removed because of acquisition failures such as incomplete Placido/OC, motion artifacts, or implausible device-derived values. These exclusions reflect technical artifacts rather than biological variation and are not systematically related to disease severity. Outliers (0.05% of excluded scans) identified by the isolation forest were predominantly morphologically extreme eyes that appeared as strong deviations in multiple indices, representing mislabeled scans (e.g., postoperative cases) and cases of highly abnormal corneal structure (e.g., corneal edema or infection), rather than borderline subclinical cases. Importantly, in routine clinical workflows, it is common practice to obtain multiple scans and rely on the best-quality image for interpretation while discarding suboptimal acquisitions. Our exclusion strategy therefore mirrors this process, suggesting minimal risk of systematic bias and limited impact on the generalizability of our findings.

Although the probability thresholds used in this study reflect standard confidence intervals,^38^ clinicians may calibrate decision boundaries based on clinical capacity or acceptable false positive rates. In our cohort, shifting the thresholds ±5% changed the number of eyes classified as SKC only modestly (±2–3%) and preserved >96% classification overlap, indicating that the SKC group is stable across reasonable threshold choices. By flagging eyes that do not meet standard diagnostic thresholds but are structurally atypical in several dimensions, this system has the potential to shift KC management from reactive treatment of overt disease to proactive surveillance and early intervention. Such an approach offers a path toward reconciling longstanding inconsistencies in the early detection of KC.
