# Supplementary material for: Data-Driven Detection of Subclinical Keratoconus via Semi-Supervised Clustering of Multidimensional Corneal Biomarkers
Source: Ophthalmol Sci. 2025 Nov 11;6(2):100998. doi: 10.1016/j.xops.2025.100998 (PMC12756640; doi:10.1016/j.xops.2025.100998)
Supplement: Supplemental Figure A [file mmc1.pdf]

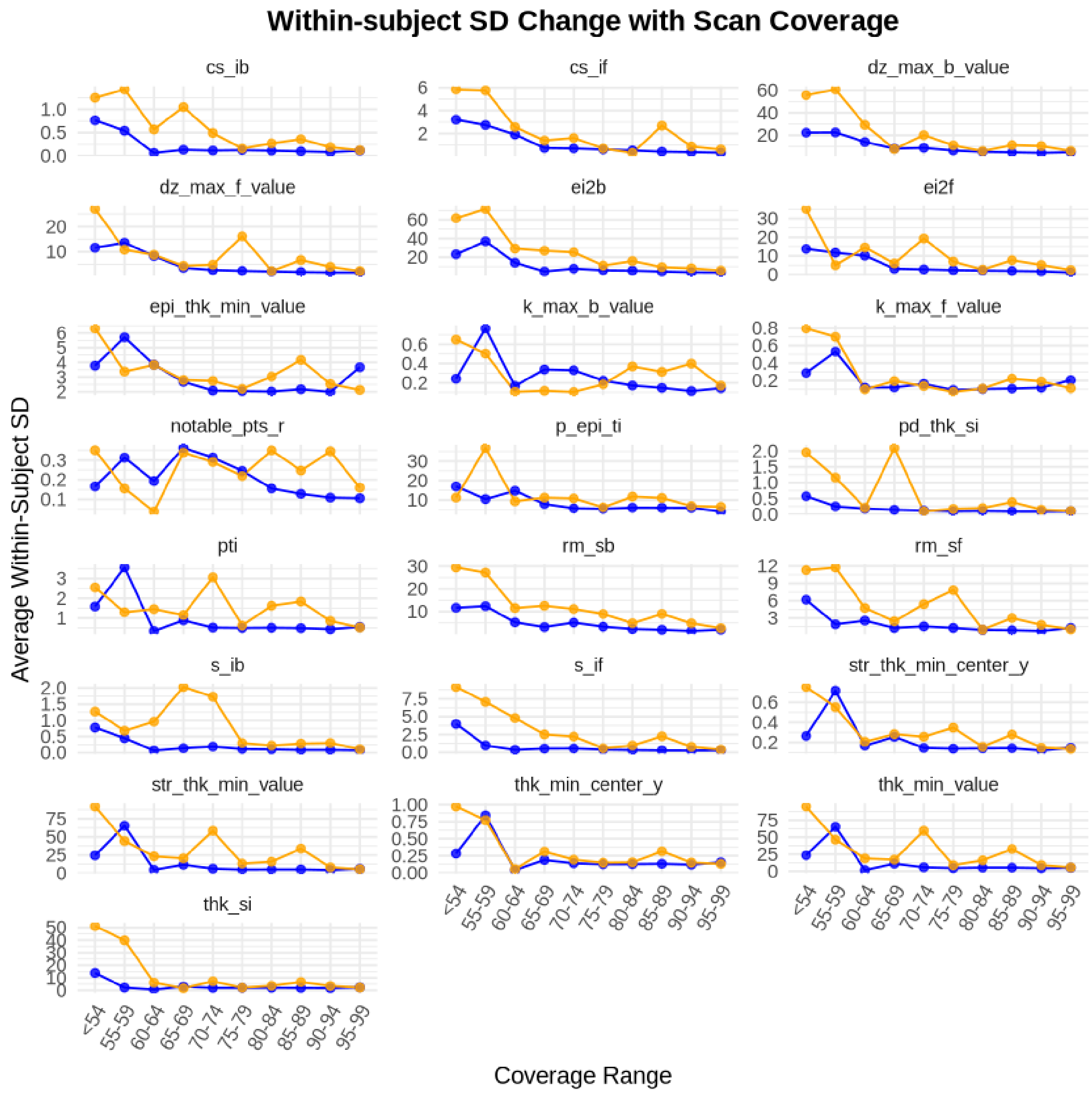

Supplementary Figure A. Within-subject standard deviation as a function of scan coverage (blue = Placido coverage, orange = B-scan coverage).
