# Supplementary material for: Data-Driven Detection of Subclinical Keratoconus via Semi-Supervised Clustering of Multidimensional Corneal Biomarkers
Source: Ophthalmol Sci. 2025 Nov 11;6(2):100998. doi: 10.1016/j.xops.2025.100998 (PMC12756640; doi:10.1016/j.xops.2025.100998)
Supplement: Supplemental Figure C [file mmc2.pdf]

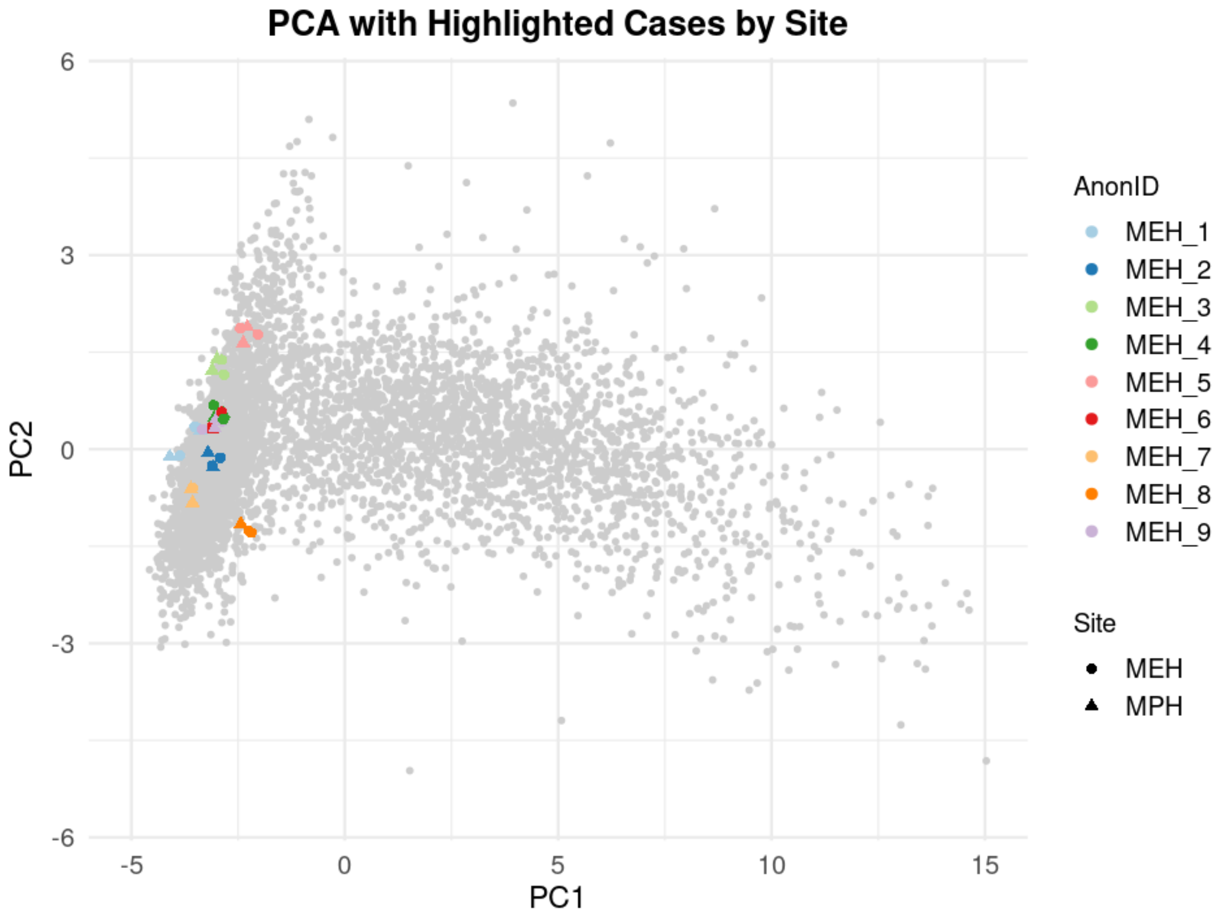

Supplementary Figure C. Paired MS-39 scans from nine healthy participants imaged at Moorfields Eye Hospital (circles) and Moorfields Private Hospital (triangles) are overlaid on the full PCA distribution of the main study cohort (grey)
