# Supplementary material for: Data-Driven Detection of Subclinical Keratoconus via Semi-Supervised Clustering of Multidimensional Corneal Biomarkers
Source: Ophthalmol Sci. 2025 Nov 11;6(2):100998. doi: 10.1016/j.xops.2025.100998 (PMC12756640; doi:10.1016/j.xops.2025.100998)
Supplement: Supplemental Figure D [file mmc3.pdf]

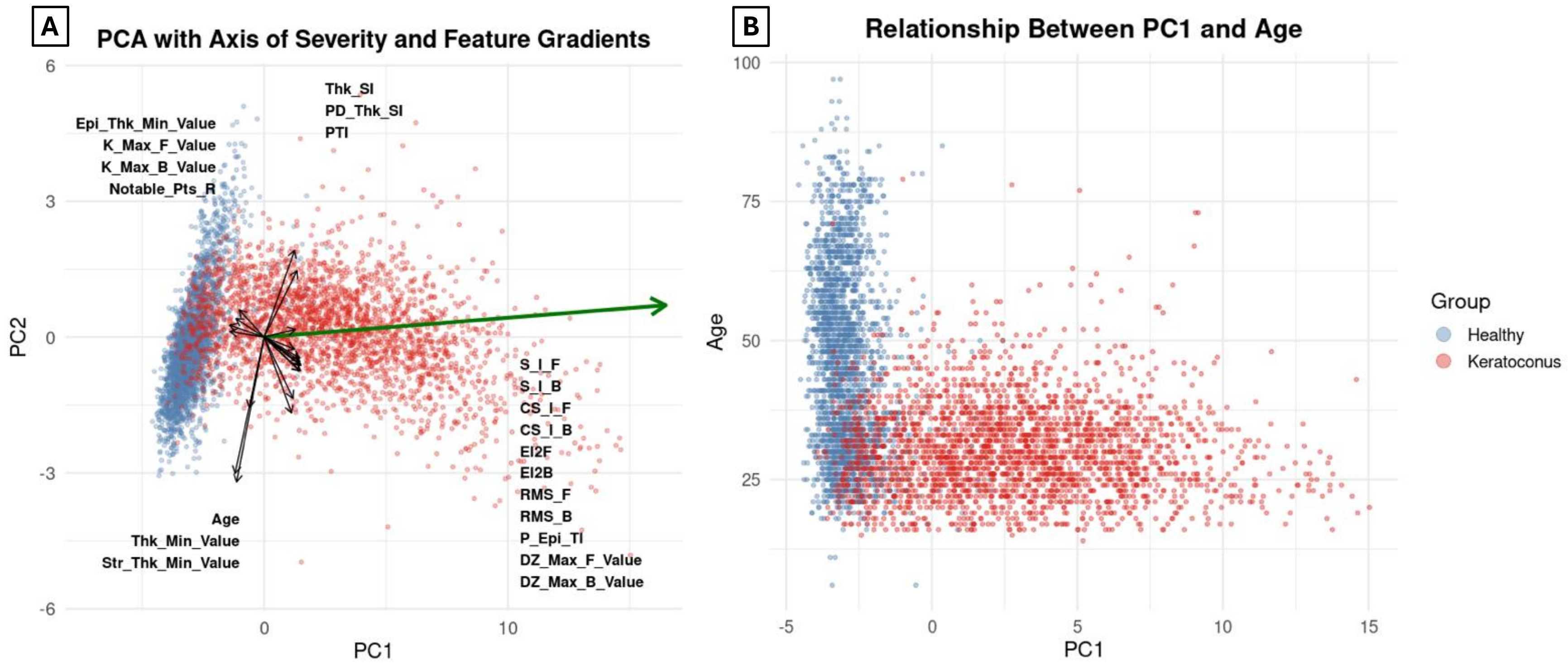

Supplementary Figure D. Principal component analysis of 21 dimensions of healthy and keratoconus eyes. Projection A demonstrates a clear separation between groups, establishing PC1 as the dominant axis of severity (green arrow), with feature vectors illustrating how each feature increases or decreases in value with respect to the PCA space (black arrows). Projection B plots PC1 values against patient age and shows that eyes with keratoconus cluster among younger patients.
