# Supplementary material for: Data-Driven Detection of Subclinical Keratoconus via Semi-Supervised Clustering of Multidimensional Corneal Biomarkers
Source: Ophthalmol Sci. 2025 Nov 11;6(2):100998. doi: 10.1016/j.xops.2025.100998 (PMC12756640; doi:10.1016/j.xops.2025.100998)
Supplement: Supplemental Figure E [file mmc4.pdf]

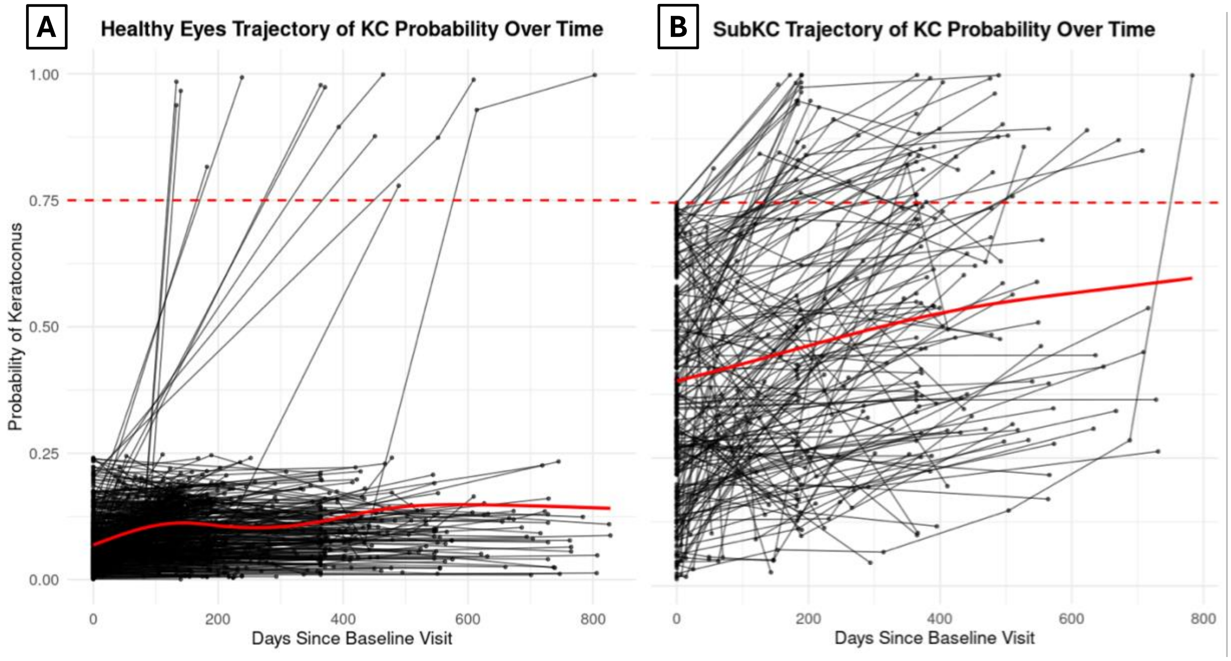

Supplementary Figure E. Longitudinal trajectory of Gaussian Mixture Model (GMM)-derived posterior probability for the keratoconus (KC) component. The solid red line represents the smoothed trend used generalized additive models, and dashed horizontal lines denote the 0.75 threshold to class conversion to KC. (A) Eyes classified as healthy showed stable KC probabilities below 25% over time. (B) Eyes classified as subclinical KC (SKC) demonstrated consistent upward drift in KC probability.
