# Supplementary material for: Data-Driven Detection of Subclinical Keratoconus via Semi-Supervised Clustering of Multidimensional Corneal Biomarkers
Source: Ophthalmol Sci. 2025 Nov 11;6(2):100998. doi: 10.1016/j.xops.2025.100998 (PMC12756640; doi:10.1016/j.xops.2025.100998)
Supplement: Supplemental Figure F [file mmc5.pdf]

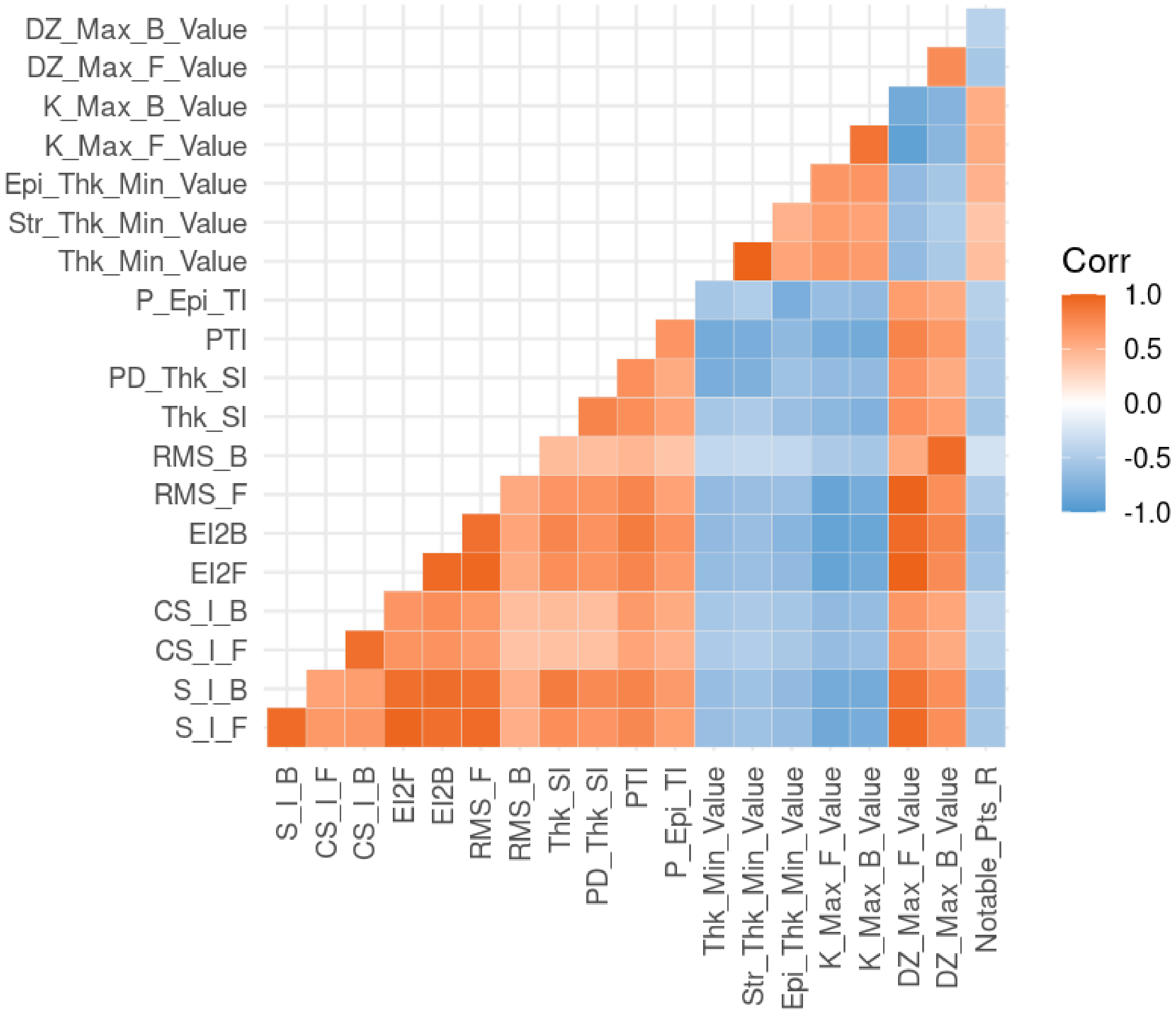

Supplementary Figure F. Pairwise Pearson correlation heatmap of selected topographic and tomographic indices used in keratoconus screening. Warmer colors indicate positive correlations, and cooler colors indicate negative correlations.
