# Supplementary material for: Data-Driven Detection of Subclinical Keratoconus via Semi-Supervised Clustering of Multidimensional Corneal Biomarkers
Source: Ophthalmol Sci. 2025 Nov 11;6(2):100998. doi: 10.1016/j.xops.2025.100998 (PMC12756640; doi:10.1016/j.xops.2025.100998)
Supplement: Supplemental Table A [file mmc6.pdf]

Supplementary Table A. Summary of biological plausibility filters applied to keratoconus-screening indices.

Feature	Cutoff	Explanation
Root Mean Square Error - Front and Back (RMSf and RMSb)	0	RMS is the square root of the averaged squared differences from a reference shape. Squared values cannot be negative.
Minimum Thickness, Minimum Stromal Thickness and Minimum Epithelial Thickness Values (ThkMin, StrThkMin, EpiThkMin)	> 0	Corneal thickness parameters measure the distance across the cross-section of the corneal layers. A negative tissue depth is impossible and would stem from an error.
Keratometry Values - Front and Back (KMaxF and KmaxB Value)	0	Keratometry measures corneal curvature in diopters, with positive values reflecting a concave shape. Negative keratometry would indicate a convex shape, which is biologically implausible.
Notable Points Radius (NotablePtsR)	0	Notable Points Radius represents the average radial distance of notable points from their barycentre. Distance and radius cannot be negative.
DZMax - Front and Back	0	DZMax is defined as the maximum positive elevation deviation from a best-fit reference surface, being positive.
