# Supplementary material for: Data-Driven Detection of Subclinical Keratoconus via Semi-Supervised Clustering of Multidimensional Corneal Biomarkers
Source: Ophthalmol Sci. 2025 Nov 11;6(2):100998. doi: 10.1016/j.xops.2025.100998 (PMC12756640; doi:10.1016/j.xops.2025.100998)
Supplement: Supplemental Table B [file mmc7.pdf]

Supplementary Table B. Proportion of variance explained by each principal component (PC) derived from Principal Component Analysis (PCA). The cumulative variance indicates the total variance captured up to and including each component.

**PC**	**Proportion Variance Explained**	**Cumulative Variance**
PC1	0.7171	0.7171
PC2	0.0625	0.7796
PC3	0.0446	0.8242
PC4	0.0414	0.8656
PC5	0.0337	0.8992
PC6	0.0256	0.9249
PC7	0.0176	0.9425
PC8	0.0145	0.9570
PC9	0.0120	0.9690
PC10	0.0074	0.9765
PC11	0.0063	0.9828
PC12	0.0044	0.9872
PC13	0.0036	0.9908
PC14	0.0028	0.9936
PC15	0.0020	0.9956
PC16	0.0017	0.9973
PC17	0.0011	0.9984
PC18	0.0007	0.9991
PC19	0.0005	0.9996
PC20	0.0003	0.9998
PC21	0.0002	1.0000
