# Supplementary material for: Data-Driven Detection of Subclinical Keratoconus via Semi-Supervised Clustering of Multidimensional Corneal Biomarkers
Source: Ophthalmol Sci. 2025 Nov 11;6(2):100998. doi: 10.1016/j.xops.2025.100998 (PMC12756640; doi:10.1016/j.xops.2025.100998)
Supplement: Supplemental Figure B [file mmc8.pdf]

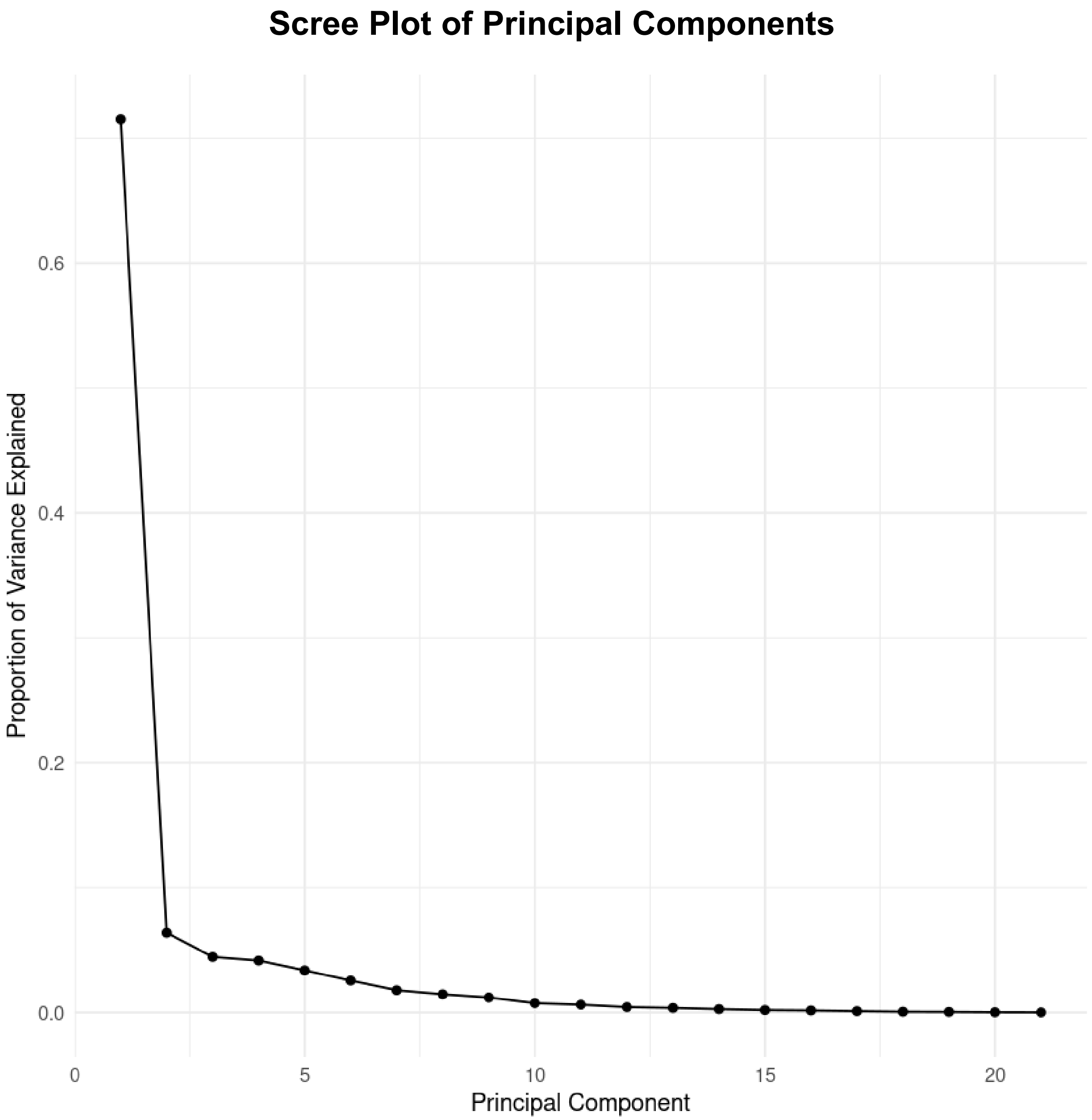

Supplementary Figure B. Scree plot (elbow method) showing the proportion of variance explained by successive principal components. The first two components together accounted for ~78% of total variance (PC1: ~70%, PC2: ~8%), while each subsequent component explained <2%, suggesting that clinically relevant variation is largely captured by the first two components.
